# The Use of Bedside Ultrasound for Gallstone Disease Care within a Community-based Emergency Department: A Confirmation Bias

**DOI:** 10.51894/001c.18182

**Published:** 2021-04-13

**Authors:** Karin H Gunther, Joshua Smith, Judith Boura, Andrew Sherman, David Siegel

**Affiliations:** 1 General Surgery Ascension Macomb-Oakland Hospital; 2 Internal Medicine Beaumont Hospital; 3 Research Ascension Macomb; 4 William Beaumont Army Medical Center; 5 General Surgery Ascension Macomb

**Keywords:** emergency department, bedside ultrasound, point-of-care ultrasound, gallbladder disease

## Abstract

**INTRODUCTION:**

Traditional evaluation for suspicion of gallstone or gallbladder-related disease includes evaluation with a formal technician-performed ultrasound. However, the use of point-of-care bedside ultrasounds (Bedside US) has been shown to be a viable alternative for the diagnosis of gallstones and gallbladder-related diseases. **Purpose Statement**: The purpose of this study was to evaluate the impact of Bedside US use in gallbladder evaluation on key patient care outcomes within our community-based emergency department setting.

**METHODS:**

This retrospective study compared the use of no ultrasound (No US), a formal technician performed ultrasound (Tech US) and Bedside US for gallstone and gallbladder related diseases within a community hospital emergency department between January 1, 2015 and January 1, 2018. Initial vitals, lab work, patient socio-demographics, medical history, emergency department length of stay in hours and disposition were reviewed.

**RESULTS:**

Of a total N = 449 patients included, patients who received a Bedside US had the fewest computerized tomography scans (No US 62% vs. Tech US 29% vs. Bedside US 16%; p < 0.0001), the shortest median emergency department length of stay (No US 4.5 days vs. Tech US 5.0 days vs. Bedside US 3.0 days; p < 0.0001), and were more likely to be discharged home (No US 41% vs. Tech US 55% vs. Bedside US 81%; p = 0.0006) compared to those that received no ultrasound or a formal ultrasound. Patients who received a Bedside US also had the statistically significant highest incidence of prior cholelithiasis (No US 29.4% vs Tech US 14.3% vs. Bedside US 31.3%; p = 0.001) and lowest total median bilirubin levels (No US 0.5 vs. Tech US 0.5 vs. Bedside US 0.3; p = 0.016) when compared to the other two groups.

**CONCLUSIONS:**

Although there was a confirmation bias, these study results indicate that point-of-care bedside ultrasound could be a viable alternative for gallstones and gallbladder-related diseases with benefits of use in a community hospital setting.

## INTRODUCTION

Gallstone disease is the most common and costly disorder affecting the body’s biliary (i.e., relating to bile or bile ducts) system.^1–3^ Gallstones are generated from an imbalance of bile salts, cholesterol and phospholipids.^2–5^ Risk factors for developing gallstone disease include female gender, age, obesity, diet, insulin-resistance, and rapid weight loss.^2,5–13^ Typically, patients first present to emergency departments (ED) with symptoms of right upper quadrant or epigastric abdominal pain, nausea, vomiting and/or jaundice.^2,13–15^

Diagnosis and evaluation for gallstone related disease begins with a complete physical exam, laboratory evaluation, and imaging with initial imaging modality of choice being an abdominal ultrasound.^15,16^ Other imaging modalities, such as computed tomography (CT) and magnetic resonance imaging (MRI), are not generally recommended as initial imaging exams. However, these modalities have proven beneficial when ultrasound results are equivocal.^17,18^

In smaller community hospital ED an ultrasound technician is not always available throughout the day to assess patients.^19^ A potential solution to this dilemma of community institutions lacking formal ultrasound technician staffing is an informal screening point-of-care bedside ultrasound (Beside US), which can be performed by ED physicians.^19,20^ The use of Bedside US has been proven beneficial in the evaluation of several other thoracic and abdominal diseases in a variety of clinical settings.^20–24^ In gallbladder diseases, Bedside US has been shown to be 55% to 95% sensitive and 82% to 100% specific for cholelithiasis (i.e., the formation of gallstones) and acute cholecystitis, as well as decreased ED length of stay.^25–34^

In rare deadly biliary diseases, such as emphysematous cholecystitis (i.e., acute infection of the gallbladder wall caused by bacterial gas-forming organisms such as *Clostridium* or *Escherichia coli*), Bedside US has been concluded to be useful in aiding identification and diagnosis.^35,36^ However, Bedside US has not been uniformly adopted in most settings despite these studies. This may be attributed to the lack of ultrasonographic training programs, technology, surgeon perception of Bedside US and the lack of high-quality prospective studies that evaluate point-of-care imaging on operative decision making in patients with cholelithiasis or cholecystitis.^19,32,34^

### Purpose of Study

The purpose of our study was to evaluate the impact of Bedside US use in gallbladder evaluation on key patient care outcomes within our community-based ED setting. The overall null hypothesis of the study authors was that they would be unable to identify any statistically significant health outcome differences across ultrasound sample subgroups.

## METHODS

The authors conducted a retrospective electronic health record (EHR) review at a Level III trauma center ED with 33,000 annual visits for patients who presented between January 1, 2015 and January 1, 2018 with symptoms for biliary disease. International Classification of Diseases, Ninth Revision, Clinical Modification (ICD-9) and Tenth Revision, Clinical Modification (ICD-10) codes pertaining to biliary disease were used by the authors to establish the initial patient list.^37,38^ Data concerning patient socio-demographics (e g., age, racial affiliation, gender identification), pertinent past medical and surgical history, allergies, initial vitals and lab results, imaging studies, length of ED stay, and ED disposition were collected by first author (KG), second author (JS), and fourth author (AS) from the Cerner Millennium® EHR system.^39^

Sample patients included those who were: 1) 18 years and older, 2) presented to the ED for the first time with signs and symptoms of biliary disease (i.e., right upper quadrant or epigastric abdominal pain) and 3) diagnosed with an ICD-9 or ICD-10 coded biliary disorder diagnosis. A total of 449 patients identified met these sampling criteria.

Patients were then categorized into: 1) those receiving no ultrasound (No US), 2) a formal ultrasound (i.e., by an ultrasound technician from the radiology department, Tech US) or 3) a Bedside US (i.e., as performed by an ED attending or ED resident physician). Before study data were collected, the study design had been approved by the authors’ Institutional Review Board at the Ascension St. John Hospital in Detroit, Michigan.

### Statistical Analyses

Descriptive statistics were first provided by the GME biostatistician third author (JB) for all data collected. Missing data remained missing and was not replaced with substitutions or imputations. All analyses were conducted by third author (JB) using SAS for Windows® 9.4, Cary, NC. Statistical significance was interpreted observing a Coefficient Alpha of 0.05 parameter.

Categorical variables are provided as counts and percent frequencies. Categorical variables were examined between the three sample subgroups with Chi-square tests where appropriate (expected frequency > five in 80% of cells), otherwise Fisher’s Exact tests were used.^40^ All continuous variables are reported in this paper as either means +/- the standard deviation or median and 25^th^ and 75^th^ percentiles, followed by the minimum to maximum dependent on the normality of the data. Continuous data was examined between the three groups with Kruskal-Wallis tests.^41^

## RESULTS

Of 449 patients evaluated, there was no statistically significant difference in patient age, gender, race, BMI, allergies (i.e. iodine), history of prior bariatric surgery, or initial vital signs between patients who received No US, Tech US or Bedside US. The only statistically significant socio-demographics or medical history difference between patient ultrasound imaging groups was in history of cholelithiasis, with patients receiving a Tech US having the lowest frequency (Table 1).

**Table 1.** Sample Socio-Demographics and Medical History

	**No Ultrasound** **(N=235)**	**Formal Ultrasound (N=182)**	**Bedside Ultrasound** **(N=32)**	**p-value**
**Age**				0.13
Mean+/- SD (median)	50+/-19 (48)	47+/-18 (45)	45+/-17 (47)	
Min to max	19 to 101	19 to 95	18 to 91	
**Gender Identity**				0.31
Males	73 (31.1%)	64 (35.2%)	7 (21.9%)	
Female	162 (68.9%)	118 (65.8%)	25 (78.1%)	
**Racial Affiliation**				0.18
White	154 (65.5%)	112 (61.2%)	16 (50.0%)	
Black	44 (18.7%)	40 (21.9%)	13 (40.6%)	
Other	7 (3.0%)	6 (3.3%)	0 (0.0%)	
Unknown	30 (12.8%)	25 (13.7%)	3 (9.4%)	
**BMI**				0.47
Mean+/- SD (median)	33+/-11 (31)	33+/-8 (32)	31+/-8 (29)	
Min to max	17 to 87	17 to 74	18 to 53	
**Medical History**				
Hypertension	95/228(41.7%)	55/178(30.9%)	10/32(31.3%)	0.067
Dyslipidemia	51/228(22.4%)	38/178(21.4%)	6/32 (18.8%)	0.89
Diabetes Mellitus	41/228(18.0%)	23/178(12.9%)	6/32 (18.8%)	0.35
Obesity	114/229(49.8%)	99/182(54.4%)	13/32 (40.6%)	0.31
Cholelithiasis	68/231 (29.4%)	25/175(14.3%)	10/32 (31.3%)	**0.001**
Prior Bariatric Surgery	16/229 (7.0%)	11/178 (6.2%)	0/32 (0.0%)	0.31
**Iodine Allergy**	7/234 (3.0%)	6/182 (3.3%)	2/32 (6.3%)	0.63

SD = standard deviation

BMI = Body Mass Index

Statistically significant values appear in **bold font**.

Pertinent labs evaluated included white blood cell count, hemoglobin, blood urea nitrogen (BUN), creatinine, total bilirubin, aspartate aminotransferase (AST), alanine aminotransferase (ALT), alkaline phosphatase (Alk Phos), and lipase. For pertinent labs, patients having a Bedside US were found to have a lower median total bilirubin value when compared to the other two subgroups (Table 2). There were no statistically significant differences found in WBC (i.e., a key indicator for acute cholecystitis) or renal function measures (i.e., BUN and creatinine). Insufficient data were available to evaluate for direct bilirubin to determine a statistical difference.

**Table 2.** Vital and lab work upon presentation

	**No Ultrasound** **(N=235)**	**Formal Ultrasound (N=182)**	**Bedside Ultrasound** **(N=32)**	**p-value**
**Initial Vitals**				
**Temperature** (Celsius)				0.14
Mean+/- SD (median)	36.8+/-0.4 (36.8)	36.8+/-0.4 (36.8)	36.7+/-0.2 (36.7)	
Min to max	36.1 to 39.6	36.1 to 39.4	36.3 to 37.1	
**Heart Rate**				0.13
Mean+/- SD (median)	82+/-18 (79)	79+/-15 (77)	80+/-17 (80)	
Min to max	49 to 163	51 to 128	48 to 136	
**Systolic Blood Pressure**				0.59
Mean+/- SD (median)	134+/-21 (134)	132+/-20 (134)	132+/-21 (134)	
Min to max	91 to 201	87 to 202	83 to 183	
**Diastolic Blood Pressure**				0.88
Mean+/- SD (median)	77+/-15 (76)	77+/-13 (78)	79+/-13 (77)	
Min to max	18 to 125	45 to 113	61 to 114	
**Respiratory rate** (breaths/min)				0.11
Mean+/- SD (median)	19+/-5 (18)	19+/-4 (18)	18+/-2 (18)	
Min to max	12 to 78	12 to 61	14 to 28	
**Labs**				
**WBC**				0.13
Mean+/- SD	10.6+/-4.1	10.2+/-4.1	9.1+/-3.0	
Median (25^th^, 75^th^)	9.8 (7.8,12.4)	9.7 (7.1,12.6)	8.7 (6.7,10.9)	
Min to max	3.4 to 33.8	3.3 to 25.1	2.5 to 16.9	
**Hemoglobin**				0.06
Mean+/- SD	13.4+/-1.6	13.5+/-1.7	13.0+/-1.6	
Median (25^th^, 75^th^)	13.3 (12.4,14.3)	13.6 (12.5,14.7)	12.9 (11.7,13.9)	
Min to max	9.6 to 18.6	4.8 to 16.9	10 to 16.6	
**BUN**				0.31
Median (25^th^, 75^th^)	12 (9,18)	12 (9,15)	12 (9,16)	
Min to max	3 to 95.5	4 to 36	5 to 22	
**Creatinine**				0.95
Median (25^th^, 75^th^)	0.79 (0.69,0.97)	0.80 (0.69,0.96)	0.81 (0.71,0.99)	
Min to max	0.44 to 3.46	0.50 to 7.20	0.49 to 1.68	
**Total Bilirubin**				**0.016**
Median (25^th^, 75^th^)	0.5 (0.3,1.2)	0.5 (0.3,1.1)	0.3 (0.2,0.5)	
Min to max	0.1 to 28.5	0.1 to 20.5	0.1 to 5.5	
**AST**				0.42
Median (25^th^, 75^th^)	27 (18,93)	28 (19,101)	25 (19,46)	
Min to max	2 to 2055	10 to 2076	12 to 467	
**ALT**				0.052
Median (25^th^, 75^th^)	27 (17,94)	31 (18,90)	22 (13,31)	
Min to max	5 to 2671	7 to 1450	7 to 230	
**Alk Phos**				0.31
Median (25^th^, 75^th^)	90 (67,144)	85 (70,130)	75 (63,113)	
Min to max	37 to 733	35 to 1601	40 to 768	
**Lipase**				0.59
Median (25^th^, 75^th^)	32 (23,49)	32 (21,48)	29 (24,40)	
Min to max	6 to 3000	10 to 30000	12 to 101	

SD = standard deviation

WBC = white blood cell

BUN = blood urea nitrogen

AST = aspartate aminotransferase

ALT = alanine aminotransferase

Alk Phos = alkaline phosphatase

Statistically significant values appear in **bold font**.

The Bedside US sample subgroup was also found to have the lowest rate of CT imaging compared with those that did not receive an ultrasound having the highest rates of CT imaging. Patients receiving a Bedside US were also found to have decreased ED length of stay and were more likely to be discharged home than patients in the other two sample cohorts (Table 3).

**Table 3:** Emergency Department (ED) Imaging, Length of Stay and Disposition

	**No Ultrasound** **(N=235)**	**Formal Ultrasound (N=182)**	**Bedside Ultrasound** **(N=32)**	**p-value**
**CT imaging**	145/235 (61.7%)	53/182 (29.1%)	5/32 (15.6%)	**<0.0001**
**ED Length of Stay (hours)**				**<0.0001**
Median (25^th^, 75^th^)	4.5 (3.0, 6.0)	5.0 (4.0, 7.0)	3.0 (2.0, 4.3)	
Min to max	1 to 11	2 to 15.5	1 to 8	
**ED Disposition**				**0.0006**
Home	96 (40.9%)	100 (55.0%)	26 (81.3%)	
Observation	10 (4.3%)	7 (3.9%)	1 (3.1%)	
Hospital admission	116 (49.4%)	64 (35.2%)	4 (12.5%)	
Transfer	13 (5.5%)	11 (6.0%)	1 (3.1%)	

## DISCUSSION

The primary benefit of a Bedside US (a.k.a. point-of-care ultrasound) remains its use in resource-limited settings, as they provide a low-cost method to aid in diagnosis and management of many conditions.^42^ Our study findings support prior findings that Bedside US may be a functional alternative to Tech US in diagnosing gallbladder-related disorders. In addition, our results resembled two previous studies that Bedside US by an ED attending or ED resident is associated with decreased ED length of stay and decreased CT scan use (i.e., unnecessary radiation exposure).^26,35^

Unlike the studies by Blaivas et al.^26^ performed at a tertiary care center or Summers et al.^32^ which was performed at an urban hospital with an emergency US fellowship, our study was conducted at a small community hospital with results more likely to be more representative of smaller institutions where ultrasound availability may be limited and the variability of patient acuity may lead to variable physician comfort levels with Bedside US use.^26,32^

As expected, the characteristics of our sample parallel the typical demographics of American patients seen with cholelithiasis and gallstone-related diseases (see Table 1): female, obese (i.e., BMI > 30), and ages within the 4^th^ and 5^th^ decades of life.^6,7,17^ However, our study results suggest that there may be a bias associated with the use of Bedside US within our smaller community hospital.

In our study, patients who received a Bedside US were more likely to have known history of cholelithiasis (Table 1), decreased total bilirubin (Table 2), and more likely to be discharged home (81.3%) than those that received a formal ultrasound (55%) or no ultrasound (40.9%) (p = 0.0006) (Table 3). Thus, our sample patients chosen to have a Bedside US in our study were more likely to have classic symptomatic cholelithiasis or biliary colic presentation than those who received Tech US or NO US. This may reflect a confirmation bias, (i.e., tendency to look for confirmatory evidence to support a diagnosis) associated with the use of the Bedside US in patients presenting with classic cholelithiasis symptoms.^43^

This conclusion is further supported by an increased disposition to discharge these patients home found within our study (81%) when Bedside US was used, as compared to an earlier study that showed only 41.7% of patients receiving a biliary Bedside US were discharged home.^35^ Unlike our study, however, this prior study was performed at a tertiary care hospital, which provided higher level of specialized medical care. Nonetheless, the expected likelihood of patients being discharged home seen in the prior study more closely reflected the percentages seen in patients receiving a Tech US or NO US (40.9% to 55%) within our study.^35^

Despite the literature demonstrating the utility of a Bedside US, the use of this modality remains restricted.^44^ The arguments against Bedside US use (e g., importance of obtaining an isolated common bile duct dilation measurement, inferiority to an accredited radiography US or limitation with non-fasting patients) have been disproven.^44,45^ However, in our study, only 7.2% of our patients received a Bedside US.

Based on review of the literature, we recommend continued incorporation of Bedside US teachings to residents and direct studies comparing the Bedside US to Tech US in complicated biliary diseases to improve confidence of Bedside US diagnosis and use.^26,33^

In 2016, the American College of Emergency Physicians has issued a statement stating that Bedside US techniques in the ED should be a fundamental element of Emergency Medicine training. Furthermore, the Accreditation Council for Graduate Medical Education (ACGME) mandated procedural competency for all Emergency Medicine residents.^46^ Although biliary is listed as one of the core ED ultrasound applications, there is still no uniform standardized implemented emergency medicine Bedside US curriculum.^47,48^

As previously mentioned, additional perceived barriers that ultimately influence surgical management include acceptance of the reliability of a Bedside US within the surgical community to influence operative decisions for cholelithiasis and cholecystitis.^28,31^ A recent 2020 survey evaluating Canadian general surgeons’ perceptions of a biliary Bedside US revealed that most surgeons believed the sensitivity of Bedside US to be much lower for cholelithiasis and cholecystitis than actual literature results.^49^

Results from a similar study mirrored the lack of confidence by general surgery and other consultants on trusting Bedside US performed by emergency medicine physicians.^50^ Meanwhile, there remains a paucity of studies in which the Bedside US was the sole modality of imaging for diagnosing biliary disease resulting in operative management.^27^

Several limitations of our study include our retrospective single-institution study design that may not be representative of the national use of Bedside US. Our analyses were also limited by the convenience of sample of patients presenting with symptoms of right upper quadrant or epigastric abdominal pain, nausea, vomiting and/or jaundice.

## CONCLUSIONS

Based on these study results, Bedside US may be a usable tool to facilitate ED evaluation of gallbladder diseases in more resource-limited community healthcare settings. However, the current practice remains to use Bedside US in a relatively selective gallbladder disease population. Further studies are needed to systematically evaluate surgeon attitudes and tailor educational interventions to address such concerns within the surgical community.
